# Endoscopic management of frontal sinus CSF leaks

**DOI:** 10.1016/j.bjorl.2020.08.002

**Published:** 2020-09-20

**Authors:** Anda Gâta, Veronica Elena Trombitas, Silviu Albu

**Affiliations:** University of Medicine and Pharmacy 'Iuliu Hațieganu' Cluj-Napoca, Department of Otorhinolaryngology, Cluj-Napoca, Romania

**Keywords:** Cerebrospinal fluid leak, Frontal sinus, Endoscopic repair, Trephination, Skull base

## Abstract

**Introduction:**

Endoscopic management of frontal sinus cerebrospinal fluid leaks has become the gold standard of treatment, with high success rates and low morbidity. The aim of this study is to review our experience in managing this challenging condition.

**Objective:**

To review our experience in treating frontal sinus cerebrospinal fluid leaks through an endonasal endoscopic approach.

**Methods:**

A retrospective evaluation of patients undergoing endoscopic surgery for frontal sinus cerebrospinal fluid leaks was performed. Demographics, defect location and etiology, surgical and reconstructive technique, complications, and postoperative followup were examined.

**Results:**

Twenty-two patients with a mean age of 40.4 years were treated surgically by the senior author between 2015 and 2019. Cerebrospinal fluid leak was either traumatic (17) or spontaneous (5). Successful first-attempt endoscopic repair was accomplished in all cases. A combined endoscopic-trephination approach was necessary in 5 patients (22.8%). No serious complications were reported, and frontal sinus drainage pathway was patent in all our cases. Revision surgery was necessary in only 2 patients for synechia formation. The mean patient followup was 22.7 months (range: 7 − 41 months).

**Conclusion:**

Progress in the field of endoscopic surgery has shifted the paradigm, establishing endoscopic repair of frontal sinus leaks as the standard of care. A few remaining limits of this approach could be addressed by combining endoscopy with frontal trephination.

## Introduction

Cerebrospinal fluid (CSF) leaks of the frontal sinus represent a challenging pathologic condition for the ENT surgeon. Traditionally, frontal CSF leaks were treated through extracranial approaches, including osteoplastic flaps, obliteration and cranialization, techniques with a high rate of significant complications.^1^ Currently, the endoscopic endonasal approach is the technique of choice in the management of frontal CSF leaks. However, the elaborate and variable anatomy of the nasofrontal outflow tract, the vital organs surrounding this structure, the narrow opening of the sinus ostium and the acute angle from the nostril to the frontal sinus all account for the technical difficulty of the endoscopic approach.^2^ Techniques of frontal sinusotomies were extensively described by Draf, ranging from anterior ethmoidectomy (Draf type I), to the extended Draf type III approach, comprising a large communication of both frontal sinuses with the nasal cavity accomplished by the removal of the intersinus septum and the frontal sinus floor.^3^, ^4^ It is widely acknowledged that frontal CSF leaks are distributed into two groups: leaks around the frontal recess, managed by means of Draf IIa or IIb techniques, and leaks of the posterior table of the frontal sinus, approached by the Draf IIb or Draf III procedures.^5^ Although the Draf III technique affords excellent visualization and instrumentation within the frontal sinus, the defect is accessible through the enlarged ostium only in 65% of patients, as recently emphasized by Becker et al.^2^ The aim of the present paper is to review our experience in managing frontal CSF leaks.

## Methods

### Study design and patient selection

After obtaining ethics committee approval (nº 107/09 Mar 2020) from the research council, the medical records of all patients diagnosed with frontal CSF leak treated by the senior author from July 2015 to August 2019 were reviewed.

**Figure 1:**
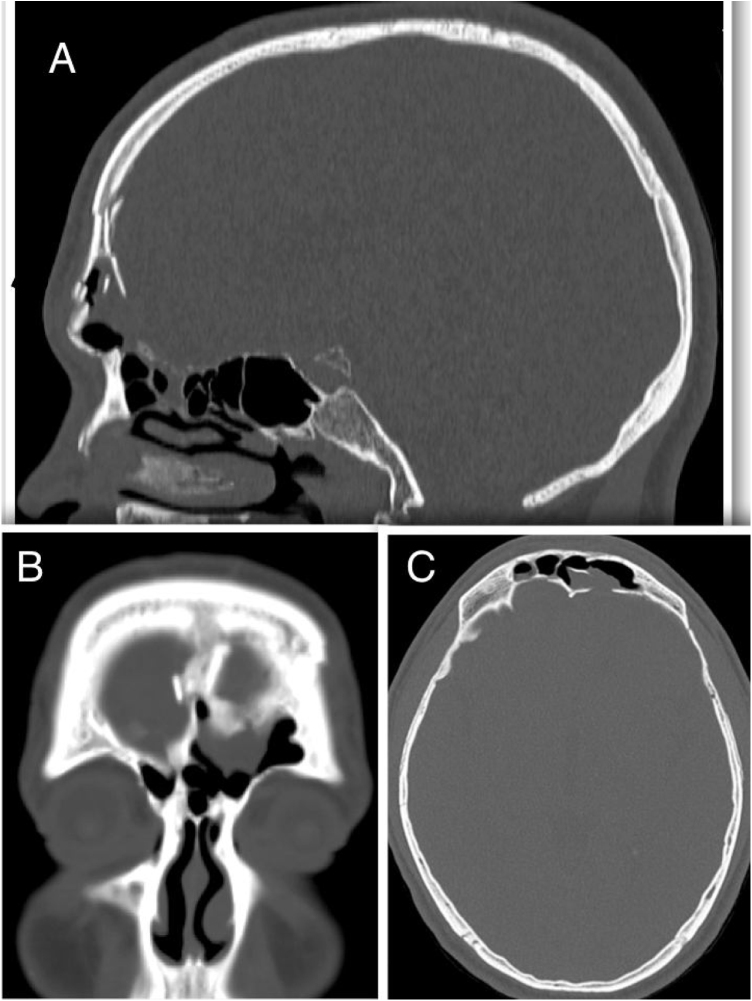
Triplanar CT scan (A, sagittal; B, coronal; C, axial planes) of a patient with traumatic CSF leak. Multiple skull base defects (frontal sinus posterior wall and ethmoid roof) are associated with anterior sinus wall fracture.

Patients with CSF leak following extensive tumor resection and trauma patients with intracranial injury requiring craniotomy were excluded. Only patients with a history of trauma or spontaneous CSF leak and a minimum of 6 months followup period were included. Demographics, history, site of the leak, surgical approach, reconstruction technique, complications and followup were retrieved from a specific database. Preoperative evaluation consisted of a history, clinical examination, thin-sliced computer tomography, magnetic resonance imaging (MRI) and β-2 transferrin assay in certain cases. The decision to perform an endoscopic approach was based on preoperative triplanar CT assessment of the defect and frontal sinus anatomy as described by Becker et al.^2^ (Fig. 1).

### Surgical approach

**Figure 2:**
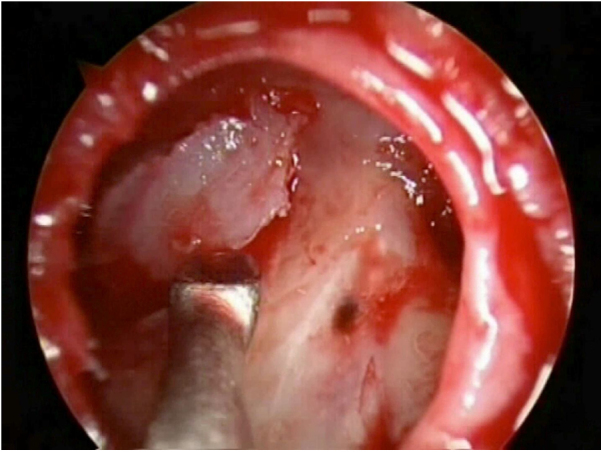
Reconstruction of defect assisted by trephination.

The patient is positioned in reverse Trendelenburg. An ephedrine-soaked wick was used to reduce nasal decongestion. Ahead of the frontal procedure, a complete sphenoethmoidectomy on the affected side is performed in order to identify any other possible leakage site and improve visualization. For defects located around the frontal sinus drainage pathway (FSDP), a Draf IIa or Draf IIb frontal sinusotomy is carried out. For defects of the posterior table, the Draf III, also known as modified endoscopic Lothrop procedure (MELP), is used. The frontal beak and intersinus septum are removed using both frontal ostium punch and Kerrison bone-cutting forceps, without making use of high-powered drills.^3^, ^4^ In cases of defects located superior or lateral on the posterior sinus wall, with a narrow anteroposterior distance of < 1 cm, or associated anterior table fractures outside of endoscopic reach, we employ the frontal trephine technique in combination with the endonasal endoscopic approach^1^, ^2^, ^6^ (Fig. 2). This combined “above and below” technique allows extensive instrumentation within the whole frontal sinus.^7^, ^8^ The incision is placed in the inferomedial brow as described by Wigand et al.,^9^ dissection is continued through all layers up to the frontal bone, with elevation of the periosteum and avoidance of the supratrochlear neurovascular bundle. The position of the trephination is determined by the location of the defect and the osteotomy is enlarged to provide surgical access for a 4-mm endoscope and surgical instrument simultaneously.

### CSF leak repair

Meningoceles or encephaloceles were reduced using bipolar electrocautery; mucosa and displaced bone fragments around the bony defect were removed and the defect was measured with a ruler before making the final decision on the appropriate graft. Reconstruction was performed using autologous materials tailored to the size and etiology of the defect. The size of the sinus defect was ascertained by the use of a curette. For small defects (less than 0.5 cm) a fat plug or free nasal mucosal grafts were used, and for larger defects the reconstruction was done usinga multilayer repair: an inlay mucosa or fascia lata graft placed intracranial, cartilage taken from the nasal septum to strengthen the first layer and fascia lata as an overlay covering the denuded bone surrounding the defect. For patients with confirmed or suspected intracranial hypertension, a septal cartilage graft was also embedded in the multi-layer technique. Abdominal fat was used to support reconstruction grafts in cases of large defects of the posterior wall. Surgicel and gelfoam were used to secure the graft into position, followed by anterior nasal packing. Lumbar CSF drains were not routinely used, except for patients with increased intracranial hypertension (ICP), given the insufficient proof of benefit.^10^ Conversely, we considered all spontaneous leaks as potential ICP. Communication with anesthesia established avoidance of preventable sudden intracranial pressure increase associated with coughing during extubation and vomiting. A perioperative prophylactic intravenous antibiotic was administered for 48 h and continued with oral antibiotics until the nasal packing was removed. In cases with spontaneous CSF leak, Acetazolamide (250 mg twice daily) was started the day after surgery and administered for one month. We followed up each patient at 1 and 3 months after surgery and biannually afterwards, with periodic endoscopic examinations.

## Results

From 2015 to 2019, a total of twenty-two patients (15 male and 7 female patients), with a mean age of 40.4 (range: 21 − 67 years), underwent surgical treatment for frontal sinus CSF leak by single ENT surgeon (S.A.). The mean follow-up was 22.7 months (range: 7 − 41 months).

A history of trauma was recorded in 17 patients (77.2%), whereas 5 patients presented spontaneous CSF leak. All patients presented with CSF rhinorheea.

The majority of patients presented with a single leak site (15 patients, 68.1%), located in the frontal recess (2 patients) or frontal sinus posterior wall (13). Multiple defects were found in 7 patients, of whom 5 were associated with an ethmoid roof defect (Table 1).

**Table 1:** Characteristics of patients included in the study.

Patient	Demography	Etiology	Defect location	Defect size (mm)	Surgical approach	Reconstruction technique	Follow-up (months)
1	M/31	Trauma	P.W.	20	Trep, IIB	Fat plug	41
2	M/42	Trauma	P.W.	40	IIB	Nasal mucosa	28
3	M/51	Trauma	P.W., Ethmoid	60	IIB	Fascia lata, cartilage	36
4	F/42	Spontan	Fr.	65	IIA	Nasal mucosa, cartilage	38
5	M/21	Trauma	P.W., Ethmoid	75	III	Fascia lata, cartilage	14
6	F/47	Trauma	P.W.	30	IIB	Nasal mucosa	10
7	F/32	Trauma	Fr., P.W.	70	IIB, trep	Fascia lata, cartilage fat graft	37
8	M/57	Trauma	P.W.	100	III	Fascia lata, cartilage	41
9	M/49	Spontan	P.W.	50	IIB	Fascia lata, cartilage, fat graft	34
10	M/67	Spontan	Fr.	35	IIA	Nasal mucosa	15
11	F/46	Spontan	P.W.	100	III	Fascia lata, cartilage, fat graft	24
12	F/40	Spontan	P.W., Ethmoid	100	III	Fascia lata, cartilage, fat graft	31
13	M/56	Trauma	P.W.	40	IIB	Nasal mucosa	26
14	M/27	Trauma	P.W.	50	IIB,trep	Fascia lata, fat graft	25
15	F/44	Trauma	Fr., P.W.	150	IIB	Fascia lata, cartilage	18
16	M/61	Trauma	P.W.	50	IIB, trep	Nasal mucosa, fat graft	17
17	M/39	Trauma	P.W.	40	IIB	Nasal mucosa	16
18	M/29	Trauma	P.W.,Ethmoid	50	IIB	Nasal mucosa	14
19	M/25	Trauma	P.W.	80	IIB. trep	Fascia lata, cartilage fat graft	12
20	M/24	Trauma	P.W.	40	IIB	Nasal mucosa	9
21	F/28	Trauma	P.W.	200	III	Fascia lata, cartilage	8
22	M/32	Trauma	Fr., Ethmoid	30	IIA	Nasal mucosa	7

P.W., Posterior Wall; Spontan, spontaneous; Fr., Frontal Recess; trep, Trephine.

**Figure 3:**
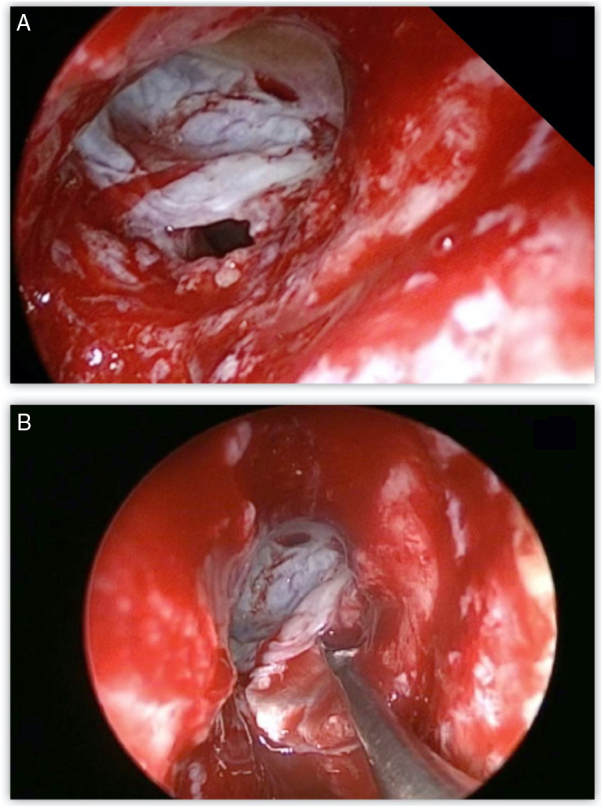
A, Dural breach associated with significant posterior wall defect visible through Draf IIB sinusotomy. B, first underlay graft in multilayer reconstruction using fascia lata.

**Figure 4:**
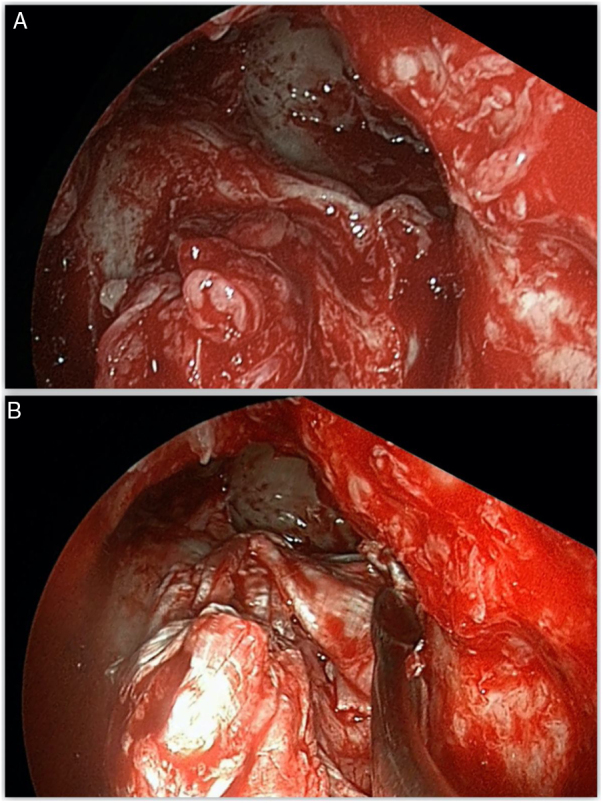
A, Posterior wall and frontal recess defect visible through Draf III sinusotomy. B, Reconstruction using fascia lata graft.

**Figure 5:**
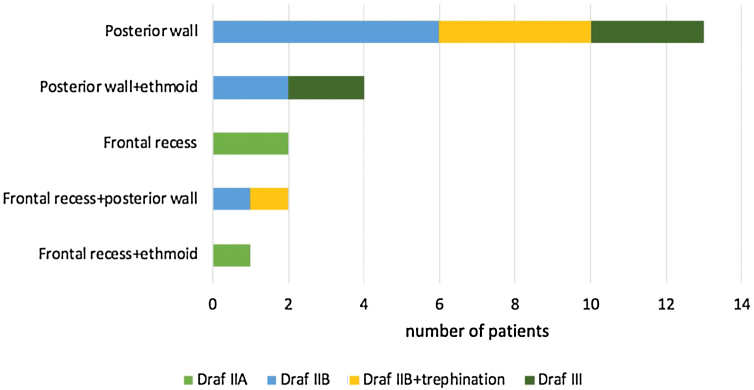
Chart corelating site of leak with surgical approach employed.

Most defects were repaired by an endoscopic approach (17 patients, 77.2%), with only 5 patients (22.8%) requiring a combined endoscopic-trephination approach. Among patients with an exclusive endoscopic approach, Draf IIB was the most common surgical technique (9 patients, 52.9%) (Fig. 3), followed by Draf III in 5 cases (29.4%) (Fig. 4), and Draf IIA in 3 cases (17.7% all of which involved the frontal recess ± ethmoid roof) (Fig. 5). All 5 cases requiring frontal sinus trephination were associated with Draf IIB sinusotomy; all patients presented a posterior wall defect and one patient associated a frontal recess leak.

**Figure 6:**
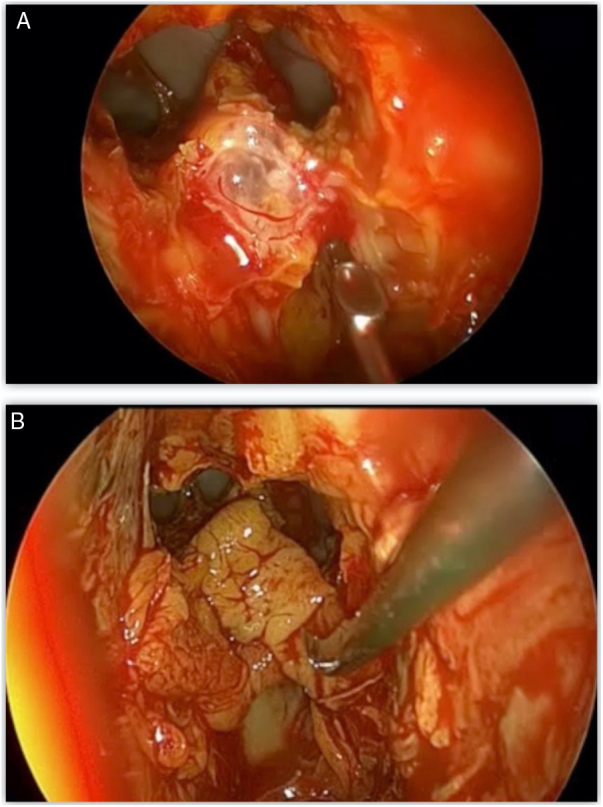
A, Defect located around the frontal recess. B, “Sandwich” reconstruction technique using septal cartilage and mucosa graft.

The skull base defect was repaired in a three-layer technique using fascia lata in 12 patients (54.5%) and 11 patients presented defects larger than 50 mm, requiring a cartilage graft. A double layer with free graft of mucoperiosteum in 9 patients (40.9%) (Fig. 6) and a single patient presented a defect small enough for a fat plug. A final support layer using abdominal fat graft was applied in 7 cases (31.8%) (Table 1).

In 22 of 22 patients (100%) successful repair was accomplished with first surgical approach and no patients experienced serious complications to date (intracranial infection, obstruction of the FSDP, mucocele formation). Clinical examination and followup CT scans demonstrated restoration of mucociliary clearance and adequate frontal sinus ventilation in all patients. One patient presented elevated intracranial pressure postoperatively, requiring temporary external lumbar CSF drainage and increase of Acetazolamide medication (250 mg, 3 times daily), without side effects. Preventive revision surgery was performed endoscopically in two asymptomatic patients presenting a synechia in the proximity of the frontal neo-ostium.

## Discussion

Frontal sinus pathology represents a challenge even for experienced endoscopic surgeons. The management of a CSF leak located in this region is associated with a high success rate on the first attempt, with low morbidity and good cosmetic results. While cranialization and osteoplastic flap provide excellent visualization and access to the frontal sinus, they require external incisions and present increased morbidity caused by cerebral edema, intracerebral hemorrhage, anosmia, frontal lobe traction, frontal lobe deficits, prolonged hospital stay and mucocele formation.^1^

### Etiology

The frontal sinus does not represent the most common site for CSF leaks. A systematic review carried out by Psaltis et al.^11^ found the ethmoid/cribriform plate to be the most common site, followed by sphenoid and frontal sinus. In a large case-series on 193 patients with CSF leaks, treated over 21 years, Banks et al.^12^ found frontal sinus defects in only 11.4% of patients. Although trauma has historically been considered the most common cause of CSF rhinorrhea, the review by Psaltis et al.^11^ including a total of 1778 fistulae repairs, has shown a higher prevalence for spontaneous CSF leaks overall. However, a study by Jahanshahi et al.^13^ reported frontal sinus CSF leaks in 24 patients, out of more than 100 patients who underwent endoscopic repair of CSF leaks. In this retrospective review, trauma was the most common etiology (18 patients), followed by spontaneous leaks (6) and without accounting for defects caused by tumor excision. Our study has similar findings, with 77.2% of CSF leaks caused by trauma and the rest being spontaneous.

### Surgical approach

All skull base defects occurring anterior to the anterior ethmoidal artery (AEA) are considered frontal sinus leaks. Becker et al.^2^ demonstrated the limitations for endoscopic visualization and instrumentation of the frontal sinus in a study performed on twenty-eight cadaver sinuses. The study revealed that a small anteroposterior diameter of the frontal recess and defects located lateral or superior on the posterior sinus wall represent the main confines.^2^ Moreover, Becker et al.^2^ ascertained no significant difference for superior visualized reach between different Draf sinusotomies.

In a study by Jones et al.,^14^ 24 patients with traumatic or spontaneous CSF leaks were treated endoscopically. Among the 24 cases, frontal sinusotomy Draf IIb was the most common approach (21 patients). An endoscopic modified Lothrop procedure was necessary in two patients and one patient underwent a combined Draf IIA sinusotomy with frontal trephination. In a study by Jahanshahi et al.^13^ on 24 patients, the surgical approach was similar, with Draf IIb being used in 20 patients, Draf III in 3 patients and Draf IIa in one patient. In the preceding author's belief,^13^ mini- upper septectomy could be performed as a low morbidity solution and Draf frontal sinusotomies are enough for replacing the trephine approach.

Frontal trephination was proposed as an adjunct to the nasal endoscopic approach and was first used in repairing CSF leaks by Purkey et al.^15^ Frontal sinus trephination proved to be an effective tool to expand the armamentarium of rhinologists and skull base surgeons facing complex frontal sinus pathology.^7^ Presently, with concerns of tissue prolapse and cosmetic deformity being uncommon,^7^, ^16^ the size of trephination can be wide enough to create a surgical corridor for the endoscope and surgical instruments, broadening endoscopic access to the frontal sinus. Frontal sinus trephination is a safe procedure with minimum risk of complications and excellent cosmetic results.^17^ The complication rate encountered with frontal trephination is as low as 6.4%, the most common being infection at the trephine site.^18^

In our study, the most common approach was Draf IIb (14 patients), followed by Draf III in 5 patients. We consider that for small defects located at the frontal recess, in the proximity of AEA, Draf IIA sinusotomy provides an adequate access (3 patients).

In a case-series of 156 patients with frontal sinus pathology, Conger et al.^19^ demonstrated an additional advantage of this combined procedure in treating frontal sinus anterior wall fractures. In two different studies, Banks et al.^1^ and Steiger et al.,^20^ managed an endoscopic stabilization of fracture by reduction of the fracture to the natural forces of interlocking segments, or by packing the sinus cavity.

In patients with frontal CSF leaks inaccessible solely by conventional frontal sinusotomy, the endoscopic endonasal orbital transposition (EEOT) procedure was recently described by Karligkiotis et al.^21^ This procedure increases exposure obtained with Draf type IIb/III sinusotomy by drilling the superomedial wall of the orbit and displacing it laterally. While Bozkurt et al.^6^ report excellent outcomes of EEOT in the treatment of selected frontal sinus CSF leaks, the technique requires advanced technology and only increases reach to the far lateral aspect of the sinus and supraorbital recess.

### Reconstruction

In a study by Shi et al.^22^ of 15 patients, reconstruction was performed with autologous materials. A “sandwich” technique was mostly used (13 patients), for 2 patients a fat plug was sufficient and the result was successful in 93% of cases after a first approach.^22^ Jones et al.^14^ reported 91.9% successful closure after a first attempt using synthetic materials as an inlay layer, vascularized septal flaps (16 patients) and free mucosal grafts as onlay.

As a result of the diverse materials and reconstruction techniques used for treatment of CSF leaks, Psaltis et al.^11^ in their literature review were not able to conclude which are ideal. However, success rates of endoscopic repair are very high, contrary to the lack of a standard reconstruction procedure. Bozkurt et al.^6^ believe that success in repairing a leak depends on suitable access, rather than the graft or reconstruction technique (Table 2). Jahanshahi et al.^13^ describe using a fat graft as the inlay layer intracranially, with a bone graft for supporting this layer in spontaneous leaks and fascia as an onlay second layer to cover denuded bone around the defect. The previous author^13^ considers that the choice of grafts and techniques are often the surgeon's personal preference. We also agree with the author^13^ that the morbidity associated with septal flaps does not always justify their use, considering the same results can be achieved using grafts. Postoperative CSF lumbar drains were not routinely used due to the scarce proof of benefit from available literature, data confirmed by a prospective study conducted at our institution, concluding that success rates of CSF leak repair are not improved with the use of lumbar drains. However we preferred resorting to the combination of lumbar drain and Acetazolamide for patients with spontaneous leaks due to the higher failure rates observed in this group.^10^

**Table 2:** Similar published clinical series on endoscopic repair of frontal sinus CSF leaks.

Study	Number of cases	Etiology	Surgical approach	Reconstruction	Outcome
Shi et al.,^22^ 2010	15	Trauma (14), spontaneous (1)	Draf IIa (9), Draf IIb (4), combined open and endoscopic (2)	Inlay muscle, onlay fascia +/- mucosa, bone in > 2 cm defects	Revision for leak (1), revision for frontal stenosis (1)
Jones et al.,^14^ 2012	24	Spontaneous (13), trauma (11)	Draf IIB(21), Draf III (3), Draf IIA + trephine (1)	Inlay synthetic, onlay mucosa, septal flap (16), bone graft for suspected ICP	Revision for leak (2), revision frontal stenosis (1)
Jahanshahi et al.,^13^ 2017	24	Trauma (18), Spontaneous (6), No tumor cases	Draf Iib (20), Draf III (3), Draf IIa (1)	Autografts double layer- Inlay fat/muscle, onlay fascia	Revision for leak (1), revision frontal (1)
Bozkurt et al.,^6^ 2019	53	Trauma (26), Iatrogenic (13), Spontaneous (14)	Endoscopic- Draf IIA (5), Draf IIB (5), Draf III (3), Draf III + orbital transposition (4) endoscopic + OPF (23), endoscopic + craniotomy(13)	autologous materials, triple-layer for large defects, double layer for small defects, gasket-seal	Revision for frontal stenosis (3)
Present study	22	Trauma (17), spontaneous (5)	Draf IIA (3), Draf IIB (9), Draf III (5), Draf IIB + trephine (5)	autologus materials, double layer for small defects, triple layer for large defects (fascia+/- cartilage for defects > 2 cm)	Revision for frontal stenosis (2)

### Outcome

The first record of endoscopic repair of frontal CSF leaks is attributed to Woodworth et al. and dates from 2005.^23^ Seven CSF leaks in six patients were successfully treated, only one patient requiring an adjuvant osteoplastic flap without obliteration. Results of endoscopic management of frontal CSF leaks have been reported successful in over 95% of cases, with low complication rates. The possibility exists of maintaining a patent frontal sinus drainage pathway and of repairing multiple skull base defects simultaneously.^1^, ^13^, ^24^, ^25^, ^26^ In a prospective case series, Jones et al.^14^ report a 91.9% success rate of closure after first attempt with an improvement up to 97.3% after subsequent endoscopic revision. The study included 37 patients treated over 3.5 years with a mean followup of 48 weeks. However, in contrast to the latter study,^14^ in our series patients with CSF leaks caused by tumors were excluded. In a retrospective case series similar in patient selection with our study, Jahanshahi et al.^13^ reported a 95.83% success rate for endoscopic repair. The case series included 24 patients with frontal CSF leaks and only one patient, an 8-year-old boy with multiple skull base fractures, developed meningitis 10 months after an apparently successful repair.

The success rate of 100% presented in our study is comparable with results from similar studies.^14^, ^19^, ^24^ Most frontal sinus CSF leaks (77.2%) were treated endoscopically. For the minority of unreachable defects, the trephination-assisted procedure managed to avoid the necessity of open approaches.

All the patients included in our study benefited from first-approach repair of the defect and the materials used for reconstruction differed from case to case, leading us to believe that that success in repairing a leak depends most on proper access of the defect and not the type of graft used. Revision surgery rates were low in the patient population and performed for minor complications (2 patients). We consider data from our study to correlate with knowledge available in the literature on this subject, supporting the routine use of endoscopic or trephination-assisted procedures for repair of frontal sinus CSF leaks, even in cases with difficult access.

The limitations of this study are similar to any retrospective review. All data included in the study was collected from patient files, detailed notes, and surgical videos. Moreover, incomplete data precluded the computation of correlations between anteroposterior diameters, size of each defect and incidence of meningoceles.

## Conclusion

Endoscopic access to the frontal sinus is considered one of the most difficult aspects of rhinologic surgery. Progress in the field of endoscopic surgery has made it the workhorse of frontal sinus surgery, gaining access even in difficult to reach areas. Endoscopic repair of frontal sinus CSF leaks is a relatively recent accomplishment and quickly has become state-of-the-art. The few remaining limitations for the use of this procedure could be addressed by combining endoscopy with the least invasive external approach, frontal trephination.

## Conflicts of interest

The authors declare no conflicts of interest.
